# Clinical Notes on Obstetric Surgery

**Published:** 1897-11

**Authors:** Joseph Eve Allen

**Affiliations:** Augusta, Ga., Professor of Obstetrics and Pediatrics in the Medical Department of the University of Georgia, Consulting Obstetrician to the City and Lamar Hospitals, Ex-President of the Augusta Academy of Medicine


### CLINICAL NOTES ON OBSTETRIC SURGERY.

By JOSEPH EVE ALLEN, M.D., AUGUSTA, GA.,

Professor of Obstetrics and Pediatrics in the Medical Department of the University of Georgia, Consulting Obstetrician to the City and Lamar Hospitals, Ex-President of the Augusta Academy of Medicine.

*Continued from the July,* 1897, *number.*

*Hydramnios.*—Dropsy of the amnion is by no means an infrequent occurrence and in the series of cases upon which these clinical notes are based was met with in forty-eight instances. This condition was repeatedly presented by the same individual and was usually attended by unmistakable evidences of constitutional syphilis. When the diagnosis is thoroughly established and pressure symptoms are present, the interests of the woman demand the termination of the pregnancy at once, for in such cases the disease so affects the fetus that it is either still-born or is not viable, and by the continuance of gestation the mother is so worn-out by prolonged suffering that she is unable to stand the strain of labor at term. The excessive distention of the uterus often brings about premature delivery, which is very apt to be followed by severe hemorrhage.

In the management of labor in these cases it should be the aim of the obstetrician to effect full dilatation of the os before rupturing the membranes, and the womb must never be emptied rapidly. The vagina should be so plugged by the hand of the operator as to cause the waters to drain off slowly. In this way time is allowed for the uterine muscular fibers to regain their vital contractility, firm, tonic retraction is assured, and hemorrhage during the third stage prevented. The extraction of the child is best accomplished by version. In several cases of hydrops amnii I have aspirated the uterus through the abdominal wall, with a view to relieving the distention and prolonging the pregnancy. In this manner as much as a gallon of fluid has been removed at one sitting without exciting uterine action. No permanent good was effected however, for the fluid was rapidly reproduced, and the state of the women soon became as bad as before the operation, necessitating the induction of premature labor. In those cases in which there is a repetition of hydrops amnii in successive pregnancies iodide of potash is often of great benefit.

*Monstrosities.*—Malformations of the fetus occurred in fifty-three cases. The most common form being that known as the exencephalous monster, in which the brain is present but the cranial bones not developed. Such cases are interesting mainly owing to the confusion in diagnosis they sometimes cause; thus, I have several times seen this condition mistaken for placenta previa, and when the *dura mater* is present, for breech presentation. In the first instance the mass filling up the cervical canal has exactly the feel of the uterine surface of the placenta, but external palpation and the absence of hemorrhage readily disclose the true state of affairs; and in the other the diagnosis is made by external palpation and the fact that the fetal genitals cannot be felt on vaginal examination.

One case of exencephalous monster that recently came under my observation is especially interesting because of its bearing upon certain operative procedures. A primipara was seen in the first stage of labor. The pains were strong and regular, but recurred at long intervals. External examination revealed the fetal head in the upper portion of the uterus and the breech presenting in the left dorso-anterior position. The cervical canal was obliterated and the external os open to about the size of a silver quarter, with the membranes protruding. Version was immediately done by the bipolar method, and as soon as the head was brought over the superior strait the bag of water was ruptured. After this the child became fixed at brim and the os quickly dilated. It was found that the face was presenting with the chin directly posteriorly. Every effort to flex the head or to rotate the chin forward was without avail, and the question of symphyseotomy was being considered, when it was noticed that the descent of the presenting part was rapidly going on and that the expected impaction did not take place. No further interference was attempted, and the child was soon born with the chin over the perineum. The mystery was then explained, for there was complete absence of skull and brain. The head was apparently implanted directly on the shoulders, there being little or no neck, hence flexion and rotation was impossible and impaction in the pelvis did not occur.

I saw once, in consultation, a case in which another kind of fetal monstrosity was mistaken for placenta previa. It was one in which there was a deficient development of the abdominal wall of the child. The child presented transversely in such a manner that the liver was forced down into the cervix, and to the touch felt exactly like placental tissue.

In this series there were several cases of absence of the extremities and of double members, such as two forearms from one elbow and many cases of spina bifida and double and single harelip, but there were no cases of joined twins or abnormally large fetus.

*Management of Breech Cases.*—In the management of breech cases the position of the woman during delivery is a matter of the greatest importance. She should always be placed across the bed in the lithotomy position, for in this position the operator is able to use both his hands to the best advantage and can promptly render such assistance as may be required. The presence of a skilled assistant is often advantageous, but anesthesia should never be resorted to except in very rare cases. When the breech is delayed in the pelvic canal I have found that the hand is generally the best instrument to effect its extraction, and that the blunt hook, fillet and forceps are of very little service. Several times I have used the forceps with good effect upon the after-coming head, and they should always be in readiness to meet an emergency. Whenever the breech presents and cephalic version is impracticable, I believe it to be good practice in all cases, as soon as the os is sufficiently dilated and before the child becomes fixed at the superior strait, to follow the advice of Dr Barnes, of London, and introduce the hand and bring down a foot, thus converting them into cases of footling presentation. This maneuver breaks the wedge, favors descent, and does not in the least diminish the mass which dilates the birth-canal for the passage of the head. It is surprising how soon, in such cases, delivery takes place after a foot has been extracted.

When the child is premature or under size, so that the head and arms can enter the pelvis together, Deventer's is the best method of delivering the after-coming head. This method consists in grasping the feet with one hand and making traction directly downward and backward, while with the fingers of the other hand pressure is made on the upper portion of the shoulders. This procedure is only suited to those cases in which the arrest is low in the pelvis or at the perineum. If the arrest is at or above the superior strait and the child is fully grown, the Prague method should first be employed, and after the head has been made to engage, the combined method should be resorted to, as it then best subserves the interests of both mother and child. In the Prague method the operator drags the feet down with one hand and the shoulders with the other until the head enters well into the pelvic canal; the body is then carried upward, while the hand on the neck prevents the descent of the occiput and thus aids in flexion. The danger in this method is that the great traction on the neck is apt to do injury to the child. This is obviated by the combined method in which flexion is accomplished by pressure upon the superior maxilla, or by pulling with two fingers in the mouth. In practising the combined method of delivery traction should never be made with the fingers in the mouth because this does not thoroughly flex the head and is very liable to lacerate the cheeks and dislocate the jaw. When delay is caused by the arms being caught up over the head, it is sometimes better to break a limb, rather than lose valuable time trying to get it down; especially is this the case when the arm is caught behind the occiput. These cases often tax the skill of the obstetrician to the utmost.

*Minor Pelvic Deformity as a Cause of Dystocia.*—Practitioners of obstetrics in the South are fortunately seldom called upon to treat cases of major pelvic deformity. Women who are natives of this section are not subject to constitutional diseases which produce those serious bone lesions that European obstetricians often meet with in their operative work. The use of the pelvimeter, however, shows that cases of minor bony distortion are with us by no means uncommon and are generally regarded as cases of uterine inertia, rigidity of the os, dry labor, etc.; the true cause of the trouble being unsuspected. The value of the pelvimeter as a means of diagnosis cannot be too earnestly insisted upon. It is to the obstetrician what the ophthalmoscope is to the ophthalmologist and the stethoscope is to the clinician. By it hidden sources of difficulty are disclosed and the proper methods of treatment pointed out. Its routine use is absolutely essential to the proper practice of modern scientific obstetrics. The reason that this instrument is not more frequently used by general practitioners is because, as formerly constructed, it was cumbersome and unwieldy and calculated to disturb and frighten patients of fine nervous organization. There is also a well-founded notion that it necessitates an indecent exposure of the person. These objections did in a measure apply to the instruments of Baudelocque and Schultze, but not to those now employed. The modern pelvimeter is light and delicate in construction, easily handled and cannot in any way offend the feelings of the most sensitive. Collyer's pelvimeter is the one that I have found the best adapted to ordinary use.

No obstetric operation should ever be attempted without the operator first gaining, by actual measurement, a thorough knowledge of the capacity of the woman's pelvis. In this way only can such mistakes be prevented as attempting delivery by forceps in cases demanding version and embryotomy when symphyseotomy would better serve the purpose.

It has been my experience that the forceps cannot deliver alive a full term fetus through a pelvic canal of less than three and three-fourths inches in the antero-posterior diameter of the superior strait. It may be sometimes possible to drag such a child through a less space, but the amount of compression to which the head is thus subjected always results in its death. By podalic version we may often safely deliver a well developed child through a pelvis that measures from three and three-fourths to three and one-fourth inches in the conjugate diameter of the brim. When the pelvis is below this size embryotomy, symphyseotomy, or the Cesarean section is necessary.

If, as should always be done, an examination is made several months prior to the time when labor is expected and a marked degree of pelvic deformity is discovered, the premature termination of the pregnancy is immediately demanded both for the sake of the mother and child. By this operation the woman is spared much suffering and danger and the life of her offspring is often saved.

*Rupture of the Uterus.*—This appalling accident occurred three times and resulted in two deaths and one recovery. All of these cases were negroes whose surroundings were the most unhygienic imaginable, and all happened before the days of aseptic midwifery. The first case was an instrumental one in which a laceration of the cervix extended up into Douglas's *cul-de-sac*, and the patient died from the combined effects of hemorrhage from the circular artery and septic infection. The next case was a primipara who had been for twenty-four hours in labor in the hands of a midwife who had administered to her repeated doses of ergot to expedite the delivery. When first seen the woman was in extreme shock. The os was fully dilated, the waters drained off and the child had partially escaped from the uterine cavity through a rent in the posterior wall. Delivery was immediately effected by the forceps and the placenta which was loose in the womb removed. Uterine contraction was perfect, but the woman never rallied from the shock and died on the second day after delivery. The third case is almost incredible and surprisingly illustrates what sometimes human nature will stand. The patient was a multipara who had been in labor attended by a midwife for about a day and a half. Two physicians were then called in, who found that the delay was due to a transverse presentation. They performed podalic version with considerable difficulty, as the amniotic sack had long ruptured and the uterus was firmly contracted on the body of the child. The delivery was effected with the exception of the head, which became impacted at the brim and resisted all efforts at extraction. I was then sent for and found the woman in shock and the child dead. I applied the cephalotribe at once and crushed the base of the skull and readily delivered. Following down the head something protruded from the vulva which at first sight seemed to be the umbilical cord, and as such, came very near being ligated and divided, but which on closer inspection proved to be a coil of small intestine. This mass had passed through a rupture in the anterior wall of the uterus. It was returned into the abdominial cavity where the placenta was discovered entirely detached and enveloped in the membranes. I was obliged to introduce my whole hand into the abdomen to remove the placenta and many large blood-clots. The woman, after the delivery, was cold, pulseless, and apparently in a dying condition. She was left as utterly hopeless, without any directions being given as to treatment, aseptic or otherwise. On the day after, to the surprise of all of us, she was still alive. Stimulants and other restoratives were then resorted to and she gradually rallied until her recovery was complete. Two of the physicians in this case have since died, but this patient is still alive, a constant reminder of the fallibility and uncertainty of professional predictions.

*Technique.*—To the obstetric, as to the general surgeon, technique is all essential to success. I therefore append to these notes a brief account of the methods I generally follow in operating, claiming for them, however, no originality but presenting them simply because experience has demonstrated to me their value. Aseptic principles of course apply as forcibly to the management of normal labors as to operative cases. In private obstetric practice all operations are usually more or less emergent in character, and hence the obstetrician, like the military and railroad surgeon, must ever be prepared to institute rigid aseptic practice under the most adverse and unfavorable conditions. In other words, he should always be independent of his surroundings and carry with him such materials and appliances as will insure his patient all the security afforded by the latest scientific methods.

As necessary to the practice of thorough asepsis the obstetric satchel should contain the following articles: Eight ounces of tincture of green soap, four ounces of alcohol, two dozen tablets of bichloride of mercury, two scrubbers in bichloride solution 1 to 1,000 contained in a screw-capped jar, four yards of plain sterile or iodoform gauze in air-tight container, two ounces of pure carbolic acid, quarter of a pound of common washing soda, creolin, and one dozen sterile towels in a metal box. The instruments should be thoroughly clean and wrapped in sterilized rolls, but should always be again boiled at the home of the patient before using. On the outside of the satchel should be strapped a long, shallow pan of enamel or granite-ware to be used as a sterilizer. Those physicians who aspire to be specialists in this branch of practice should carry their instruments, needles, suture material and dressings packed in some one of the portable sterilizers sold for the purpose. Of these, Booth's is perhaps the best.

Furbringer's method for making the hands aseptic is the simplest and easiest, and therefore the one best suited to obstetric practice. It consists in first cleaning the nails with a metal nail scraper, then scrubbing the hands for at least three minutes with tincture of green soap and boiled hot water, using a sterilized brush and changing the water three or four times during the process. The hands are then rinsed in 95 per cent. alcohol and afterwards soaked for one minute in 1 to 1000 bichloride solution. While operating, the obstetrician should wear a rubber apron to protect his clothing and over this a freshly sterilized gown to keep his hands from becoming infected. The patient is prepared, when time and the occasion will permit, by the nurse giving her a full warm bath, and in every case the field of operation should be rendered aseptic by scrubbing the external genitals, thighs and abdomen, with tincture of green soap and hot boiled water and then using alcohol and bichloride solution as in disinfecting the hands. Antiseptic douches are not necessary and should never be employed unless there is reason to suspect that the vagina has become infected; in which case this canal should be first thoroughly washed out with sterile normal salt solution and then scrubbed with tincture of green soap by means of pledgets of iodoform gauze held in long dressing-forceps, the parts being widely separated by the fingers or a Sims speculum, after which another douche of normal salt solution should be given. Bichloride solutions should never be used in the birth-canal because they coagulate the secretions and thus make the labor difficult. They are no more effective than certain other antiseptics.

Lubricants are now well-nigh things of the past in the treatment of labor cases, for it has been proven that in the great majority of instances the mucus that is so freely poured out at this time is all that is required to facilitate the passage of the child through the maternal structures. If a lubricant seems to be necessary, some sterile material should be used, such as boiled sweet oil or a 5 per cent. creolin solution. Under no circumstances must lard ever be employed.

Metal instruments, silk and silkworm gut, glass douche tubes, etc., are sterilized by boiling for half an hour in a 1 to 5 per cent. solution of washing soda. Needles, scissors and clamps are made aseptic by immersing in pure carbolic acid. Catgut should never be used.

The obstetrician should ever exercise a watchful supervision over the actions of the nurse, for if she has shortcomings they will certainly vitiate the most elaborate technique. She must always scrupulously observe all of the aseptic precautions practiced by the medical attendant, for if she touches the patient with dirty hands she is just as liable to carry infection. The patient should be especially cautioned against examining and handling herself, for in this way blood-poisoning is often produced. I have seen several cases where infection was directly due to this cause. When strict attention is paid to these matters of detail, a favorable result will often be attained, even in very unpromising cases and despite the most unfavorable environment.

The advance of practical medicine is only accomplished by the record and classification of personal experiences. These clinical notes are therefore given to the profession with the hope that they may be interesting and perhaps contain something of value to those who are especially engaged in obstetric work.

